# Moving beyond Type I and Type II neuron types

**DOI:** 10.12688/f1000research.2-19.v1

**Published:** 2013-01-22

**Authors:** Frances K Skinner

**Affiliations:** 1 Toronto Western Research Institute, University Health Network, Toronto, ONT, Canada; 2 Departments of Medicine (Neurology) and Physiology, University of Toronto, Toronto, ONT, Canada

## Abstract

In 1948, Hodgkin delineated different classes of axonal firing.  This has been mathematically translated allowing insight and understanding to emerge.  As such, the terminology of ‘Type I’ and ‘Type II’ neurons is commonplace in the Neuroscience literature today.  Theoretical insights have helped us realize that, for example, network synchronization depends on whether neurons are Type I or Type II.  Mathematical models are precise with analyses (considering Type I/II aspects), but experimentally, the distinction can be less clear.  On the other hand, experiments are becoming more sophisticated in terms of distinguishing and manipulating particular cell types but are limited in terms of being able to consider network aspects simultaneously.   Although there is much work going on mathematically and experimentally, in my opinion it is becoming common that models are either superficially linked with experiment or not described in enough detail to appreciate the biological context.  Overall, we all suffer in terms of impeding our understanding of brain networks and applying our understanding to neurological disease.  I suggest that more modelers become familiar with experimental details and that more experimentalists appreciate modeling assumptions. In other words, we need to move beyond our comfort zones.

## Introduction

Current research in my group involves developing, using and analyzing models of neurons and networks in hippocampus. Two aspects relating to the Society for Neuroscience meeting 2012 in New Orleans provided the final push for me to put my thoughts into writing via this opinion piece. First, when being questioned at one of our posters involving fast-spiking inhibitory cell models, we were asked, not for the first time, whether our neuron was Type I or II. Second, a symposium write-up in the *Journal of Neuroscience* ^1^ stated that “…fast-spiking inhibitory cells… have a hard Class 2 threshold…”. On the face of it, these are reasonable questions and statements given the theoretical basis of Type I/II neurons ^2^. However, on further reflection, I think that such questions and statements may be obscuring the hard challenges and unintentionally developing a divide by focusing on the theory rather than the theory together with the biology.

## History and hope

Hodgkin’s (1948) ^3^ classification of repetitive firing of axons into three types is common knowledge in the Neuroscience community. Specifically, the first two ‘classes’ are commonly referred to as Type I and II, where Type I neurons are able to exhibit arbitrarily slow frequencies as injected current levels are reduced, unlike Type II neurons, which cannot. In turn, these Types can be ‘translated’ to dynamical systems terminology as saddle node on an invariant circle and Andronov-Hopf type bifurcations respectively ^2^. Such theoretical interpretations have allowed insight and understanding of the control of axonal firing to be obtained, including the annihilation of firing by appropriately timed inputs ^4^. Additional theoretical aspects using Type I/II neurons have been developed. For example, Ermentrout (1996) ^5^ has shown that a general property of Type I neurons is that they have phase response curves (PRCs) that are strictly positive. This means that depolarizing stimuli given at any time point of the firing (oscillating) neuron will always lead to an earlier start of the next action potential. This has subsequent implications for the ability of Type I or II neurons to synchronize with excitatory or inhibitory connections. In essence, whether a neuron is Type I or II endows it with different neuro-computational properties. Type I neurons, so called integrators can encode input strength, whereas Type II neurons, so-called resonators, cannot. However, Type II neurons can exhibit subthreshold oscillations, whereas Type I neurons cannot. These and many other interesting aspects are detailed in Izhikevich’s book ^2^. He envisions a research program in which one is not only concerned with a neuron’s details (ion channels etc., as could be explored by experimentalists) but also with its neuro-computational features or what kind of bifurcations it expresses (as could be explored by mathematicians). I agree with this view. Indeed, he states that one of the goals of his book (p.20) is to “…bring these two groups of people closer together”. But…

## Problem and challenge

Cellular and synaptic biological details are of course important in the functioning brain, but possibly not all details are critical in all contexts. The expanding field of Connectomics is providing much information about synaptic and architectural details that can be used in computational modeling studies ^6^. For cellular aspects, using Type I and II neuron models and their PRC characteristics has been most helpful. For example, Stiefel and Gutkin (2012) ^7^ have described how different acetylcholine levels could lead to switching of cortical, pyramidal cells between Type I and II due to modulating biophysical characteristics in cellular models. Based on previous theoretical studies, this would have effects on network synchrony. A switching of PRC characteristics (in terms of Type I/II) with carbachol has been shown experimentally for Layer 2/3 pyramidal cells. These experimental studies were done *in vitro* which represents a different synaptic network environment than *in vivo* ^8^. Prescott et al. (2008) ^9^ created an *in vivo*-like environment in the dish and compared model and experimental work to indicate that CA1 hippocampal pyramidal cells switch from integrators (Type I) to resonators (Type II) when moving from *in vitro* to *in vivo*. The modeling studies implicated an M-type potassium current underlying the Type I/II switching.

I would like to highlight two issues that these studies bring forth. First, these cellular-based studies assume that Type I/II differences are important in network synchrony based on prior theoretical studies. It is of note that these differences have now been fully examined in excitatory networks ^10^ where network structure, synaptic strength and firing frequencies were examined. The underlying assumption of course is that network synchrony (such as at gamma frequencies) is important in cognitive functioning. Unlike in several invertebrate systems – such as the network controlling the movement of teeth in the stomach of crustaceans ^11^ – the function of the neuronal network can be speculative. Second, whether a mathematical model representing a neuron is Type I or II can typically be unambiguously determined using bifurcation analyses. However, whether a neuron in experimental work can be identified as being Type I or II is a bit trickier as the resolution of the injected current as well as the length of time for which the current is injected would affect the resulting frequency-current curve. Tateno et al (2004) ^12^ clearly showed a difference for fast-spiking inhibitory cells (Type II) and regular spiking pyramidal cells (Type I) in rat somatosensory cortex, which underlies the statement of “… hard Class 2” in ^1^. Whether one should extrapolate to fast-spiking inhibitory interneurons as being Type II in general is potentially not appropriate as recent studies would seem to indicate that fast-spiking inhibitory interneurons in hippocampus could be Type I, see Figure 6 in ^13^. As stated at the beginning of this section, knowing whether a neuron is Type I or II is helpful. However, this may not be the essential aspect when the experimental context and biological specifics are also a focus. For example, Sritharan and Skinner (2012) ^14^ used a previously developed biophysical model of a hippocampal interneuron (with Type I-like characteristics) to examine spike reliability. They found that spike reliability at theta frequencies was favoured by an *in vivo*-like environment that emphasized large inhibitory, but not excitatory, fluctuations. While this did not specifically depend on Type I-like model neurons, the results were more prevalent with Type I-like model neurons. In an earlier study, Tateno and Robinson (2006) ^15^ used dynamic clamp to examine Type I (regular spiking) and Type II (fast-spiking) neurons under various conditions involving fluctuating synaptic inputs. They found that while Type I and II aspects still gave rise to differences, this was not always the case. For example, in the context of relative timing of excitatory and inhibitory inputs, as could be important in cortical columnar circuitries, spike reliability and jitter were similar for both types.

As neuroscientists, we want to understand the workings of our brains and nervous systems, and it is clear that mathematical modeling and theoretical concepts are key to advancing our understanding. However, today, it sometimes feels as if we are trying too hard to fit the biology to the theory rather than using the theory to help us understand the biology/experiment. Indeed, theoretical and modeling studies provide much needed guidance, and consideration of Type I/II neurons has, and will continue to provide such guidance. However – to borrow a statement from the Society for Neuroscience meeting 2012 – we need to ‘embrace the messiness’ (as stated by Larry Abbott in his Albert and Ellen Grass Lecture “The Collective Wisdom of Neurons”). This interesting talk emphasized having some readout from the biological circuit from which we could come up with reduced descriptions and models to help us understand the system. He pointed out that although we are making progress in determining readouts, progress is limited in linking the reduced circuit models back to the biology. In my opinion, this limited progress may be partly due to insufficient interactions between theoretical, modeling and experimental studies so that the hard but essential questions are less likely to be asked. This is the problem. In the context of what I have presented here, although the dichotomy of Type I and II neurons has been immensely insightful, we need to move beyond this to also be clear about experimental context and biological specifics wherever possible. This is the challenge.

## What to do?

For theoretical analyses, mathematical models need to be in hand. In order for them to be in hand, experimental data has to be consulted. Exactly what data, how much, at what level, and how to access immediately come to the fore. Many different models have been employed in examining Type I and Type II neurons, but if the goal is to get to the heart of the different spike-generating mechanisms exhibited by Type I or II neurons, then clearly one needs to get into spatial aspects and biophysical details. For example, see ^16^. However, to understand how and why these detailed differences may be important, a clear context (‘readout’) is needed. Unfortunately, often this is either not possible and/or very difficult to obtain and/or speculative and unclear. As such, cellular-based network models have been used in different ways ^17^. Whether one is focused mainly on readouts or on cellular models, they ultimately need to be linked in some way. How best to go about it is far from clear, but in my opinion (and borrowing a well-known quote), “Resistance is futile”. Thus, what to do?

First of all, there should clearly never be any confusion or conflict when comparing modeling or theoretical studies given that model specifics, assumptions and limitations can always be spelled out. Next, I think that we all need to move beyond our comfort zones more often. That is, modeling studies should make more linkages with experimental studies, suggesting specific and particular next steps so as to help ‘get the conversation started’. However, to do this, the practicalities and limitations of experimental studies need to be considered by more modelers (e.g., is there too much variability in the experiment to apply the modeling/theoretical insights? How do model parameters relate to experimental measurements being performed? How are the experiments performed?). Also, experimentalists need to read beyond the model results, questioning and understanding model assumptions and theoretical limitations so that one can assess if and how the model insights might apply and be interpreted in particular experimental and biological situations. There are clearly practical challenges in moving beyond one’s comfort zone, but for the greater good (of tackling the immense challenges of understanding brain workings and neurological disease), I think that this needs to be done on a regular basis. While combined efforts require patience, open-mindedness and respect for the realities of different research environments, we should perhaps also try to create more opportunities for “antedisciplinary” science ^18^.

## In closing

Recently, I was excited reading an “experimentally inspired theoretical study” ^19^ in which theoretical insights from Rall ^20^ were used to shed light on biophysical design specifics. Besides my own interest in the details of the work, I was happy to see this work given what we know about the challenges faced by Rall in his day when he combined theoretical and experimental work. While I have focused on Type I/II issues and examples here, my opinion applies more widely, so that I end this opinion piece with a similar statement used before – “Neither ignore the details nor be consumed” by them ^21^. Let’s move beyond our comfort zones together.
