# Supplementary material for: Moving beyond Type I and Type II neuron types
Source: F1000Res. 2013 Jan 22;2:19. [Version 1] doi: 10.12688/f1000research.2-19.v1 (PMC3752709; doi:10.12688/f1000research.2-19.v1)
Supplement: Peer review history

# Peer review: Referee response for version 1

**Published:** 2013-02-13

**Authors:** Joel Tabak

**Affiliations:** Biomedical Research Facility, Florida State University, Tallahassee, FL, USA

I have read this submission. I believe that I have an appropriate level of expertise to confirm that it is of an acceptable scientific standard.

# Peer review: Referee response for version 1

**Published:** 2013-01-29

**Authors:** Horacio Rotstein

**Affiliations:** Department of Mathematical Sciences, New Jersey Institute of Technology (NJIT), Newark, NJ, USA

I think the paper is timely and interesting, here are my comments:

- Page 2, column 1: "In turn, these types can be 'translated' to dynamical systems terminology."
  To say 'translated' is to imply that dynamical systems tools provide an alternative (and equivalent) description of the dynamics. I don't think this is correct. I think dynamical systems analysis provide a mechanistic explanation of the dynamics in the form of a description of what causes the dynamics. 
  Also, the dynamical systems description is correct in 2D. Other dynamics may occur in 3D (e.g., canards that may lead to arbitrarily slow frequencies with an underlying Hopf bifurcation. 
  Finally, 2D saddle-node bifurcation (away from an invariant circle) can generate type II dynamics, at least theoretically. 
- Page 2, column 1: "Phase response curves that are always positive."
  Although it is probably implicit in the text that follows, it should be noted whether these PRCs correspond to excitation or inhibition.  
- The classification of type I neurons as integrators and type II neurons as resonators is far from clear to me. In fact, I am not sure that the concept of integrators and resonators is well defined with a definition that has a broad consensus. I might be mistaken, but I think it would be better to leave all that as open questions.
- I'm not sure I agree with the statement in "Second,..." (page 2, column 2). I think this is true for 2D systems, but not for higher-dimensional systems. 
- I strongly agree with the sentence that follows and I think this should be highlighted. 

- I think that, with appropriate modifications, the sentence at the end of column 2, page 2 "As stated..." should be the first sentence in the paragraph. 

I have read this submission. I believe that I have an appropriate level of expertise to confirm that it is of an acceptable scientific standard.

# Response

**Published:** 2013-02-21

**Authors:** Frances Skinner

**Affiliations:** University Health Network, Canada

All points made by the referee are well-taken.  Thank you!

I respond more specifically below - the numbering is for the 6 bullet points of the referee.

1) I completely agree that dynamical systems analyses is about trying to obtain a mechanistic explanation and not just an alternative description.  Translated in quotes was used loosely here. 2D was assumed but not explicitly stated as the referee correctly points out.

2) Yes, the assumption is that it corresponds to excitation .

3) The referee has hit upon an essential aspect that I intended to bring forth in this opinion.  That is, while mathematical models can be well-defined, biological aspects can only be well-defined when (enough) details and context are also provided.  This is what I find is often the main challenge in combining experimental work and mathematical modeling.

4) The underlying assumption here again was 2D.  Thanks for bringing this to the fore.

5) We're on the same page here!

6) While I agree that such modifications could be done, after some thought, I think that it will require quite a bit of readjustment of the flow of the entire 'Problem and Challenge' section.  I hope that the referee comments and my response will help readers further hone in to the essential point being made.  

That is, while Type I and II aspects can be helpful, they shouldn't become the focal point of our attempts to understand the biological system (as it may not be an important difference in a given biological context).  Rather, Type I/II aspects should be considered as a potentially helpful stepping stone.

# Peer review: Referee response for version 1

**Published:** 2013-01-24

**Authors:** Ernest Barreto

**Affiliations:** School of Physics, Astronomy, and Computational Sciences, George Mason University, Fairfax, VA, USA

I have read this submission. I believe that I have an appropriate level of expertise to confirm that it is of an acceptable scientific standard.
